# Constructing an Indoor Floor Plan Using Crowdsourcing Based on Magnetic Fingerprinting

**DOI:** 10.3390/s17112678

**Published:** 2017-11-20

**Authors:** Haiyong Luo, Fang Zhao, Mengling Jiang, Hao Ma, Yuexia Zhang

**Affiliations:** 1 Beijing Key Laboratory of Mobile Computing and Pervasive Device, Institute of Computing Technology Chinese Academy of Sciences, Beijing 100190, China; 2 School of Software Engineering, Beijing University of Posts and Telecommunications, Beijing 100876, China; zfsse@bupt.edu.cn (F.Z.); jiangmengling@bupt.edu.cn (M.J.); mahao@bupt.edu.cn (H.M.); 3 School of Information and Telecommunication Engineering, Beijing Information Science and Technology University, Beijing 100101, China; zhangyuexia@bistu.edu.cn

**Keywords:** indoor localization, floor plan construction, crowdsourcing, affinity propagation clustering, DTW

## Abstract

A large number of indoor positioning systems have recently been developed to cater for various location-based services. Indoor maps are a prerequisite of such indoor positioning systems; however, indoor maps are currently non-existent for most indoor environments. Construction of an indoor map by external experts excludes quick deployment and prevents widespread utilization of indoor localization systems. Here, we propose an algorithm for the automatic construction of an indoor floor plan, together with a magnetic fingerprint map of unmapped buildings using crowdsourced smartphone data. For floor plan construction, our system combines the use of dead reckoning technology, an observation model with geomagnetic signals, and trajectory fusion based on an affinity propagation algorithm. To obtain the indoor paths, the magnetic trajectory data obtained through crowdsourcing were first clustered using dynamic time warping similarity criteria. The trajectories were inferred from odometry tracing, and those belonging to the same cluster in the magnetic trajectory domain were then fused. Fusing these data effectively eliminates the inherent tracking errors originating from noisy sensors; as a result, we obtained highly accurate indoor paths. One advantage of our system is that no additional hardware such as a laser rangefinder or wheel encoder is required. Experimental results demonstrate that our proposed algorithm successfully constructs indoor floor plans with 0.48 m accuracy, which could benefit location-based services which lack indoor maps.

## 1. Introduction

During the last decade, the number of smartphones and mobile devices has increased rapidly, and various location-based applications have arisen, such as location-enabled social networking, navigating, and advertising.

As a prerequisite of these location-based applications, outdoor maps are now available for almost all regions around the globe. In contrast to this ubiquitous coverage, the coverage of indoor areas (floor plans) remains sporadic, hindering the spread of indoor location-based applications.

The traditional method of hiring professional staff to construct an indoor map inch by inch is expensive and time-consuming, and cannot be applied to large-scale indoor coverage. Furthermore, the occasional change of indoor floor plans poses a challenge to the updating of indoor maps within a reasonable timeframe.

In recent years, crowdsourcing has been applied to constructing and updating indoor floor plans. These crowdsourcing floor plan construction methods resort to anonymous users to measure indoor environments. The core idea behind constructing floor plans using crowdsourcing is aggregation of multiple mobility traces. Each mobility trace is inferred from inertial measurements, before combining user mobility traces to derive the approximate shape of accessible areas of floor plans, e.g., hallway/room shape and connectivity of floor plans. To eliminate error accumulation during dead reckoning, a small number of anchor points (e.g., entrances/exits of elevators/escalators/stairs and locations with GPS reception) with unique sensing data signatures are used to correct the drift in mobile traces. However, in many indoor environments, such anchor points are too sparse to provide sufficient and accurate correction. Therefore, both over- and underestimation of accessible areas easily occurs, e.g., when a trace drifts into walls, or when corners exist that users seldom walk around.

Here, we propose an improved algorithm for indoor floor plan construction based on magnetic fingerprinting. Our algorithm leverages extensive crowdsourcing data from multiple mobile users to construct the floor plans of indoor environments.

Our proposed method combines geomagnetic matching and dead reckoning techniques, and uses trajectory clustering and graphics processing methods to build relatively accurate floor plans. We use trajectory clustering techniques to group the trajectories inferred from different walking paths with similar geomagnetic sequences for further fusion to extract geometric features (e.g., widths of store entrances, lengths and orientations of adjoining walls) of individual landmarks from images.

The main contributions of our work are briefly summarized as follows:

(1) Our method employs indoor steady magnetic sequence data to construct the layout of indoor environments.
(2) We designed a multiple-trajectory-snippet fusion approach based on DTW (Dynamic Time Warping) match to reduce accumulation errors originating from noisy sensors, thus obtaining accurate indoor path estimations.
(3) Our proposed system is convenient to deploy and does not rely on any additional infrastructure. The mobile sensing mechanism allows our system to represent the layout of buildings. As more users collaborate, enhanced resolutions of the maps are obtained. Our proposed algorithm opens up the possibility of fast and scalable floor plan reconstruction.

This paper is organized as follows: In Section 2, we discuss previous work on indoor localization and related approaches. In Section 3, we detail the proposed crowdsourcing indoor floor plan construction algorithm based on magnetic fingerprint. In Section 4, we evaluate the proposed scheme, and in Section 5, we conclude the paper and discuss future work.

## 2. Related Work

Much research over recent years has addressed indoor localization, including the use of radio frequency (RF) [1,2,3,4,5], magnetic signals [6,7,8], computer vision [9,10], infrared signals [11], ultrasonic signals [12], and dead reckoning methods [13]. These approaches usually assume the existence of building floor plans.

Recently, crowdsourcing has been used for both outdoor and indoor localization. These approaches focused on constructing an RF fingerprint database [14], while other designs use the dead reckoning approach by accessing data from accelerometers, gyroscopes, and compasses to generate the trajectories of users and reconstruct indoor floor plans. For instance, to enhance the odometry trace accuracy, CrowdInside [15] analyzed the sensing data of unique indoor anchor points (e.g., building entrances, elevators, stairs, and escalators) for calibrating the accumulated motion trace errors. In comparison, MapGenie [16] used mobile trajectories inferred from the foot-mounted IMU (Inertial Measurement Unit), which is more accurate in step length and heading estimation; however, this method requires special hardware and is inconvenient to use.

Other approaches used Wi-Fi signatures to discover the layout of buildings. Jiang et al. [17] proposed several algorithms that evaluate the Wi-Fi signature similarities between different rooms and hallway segments to identify their locations relative to each other, and combine the inertial data to estimate the hallway lengths and orientations for constructing the indoor floor plans. However, this method requires manual association between the Wi-Fi fingerprints and corresponding room IDs. In another work, Walkie-Markie [18] was used to identify the locations where the trend of Wi-Fi signal strength reverses direction, and leverages these stable locations as anchor points. SmartSLAM [19], a smartphone-based indoor SLAM (Simultaneous Localization and Mapping) solution employing the Bayesian estimation method, was used to construct an indoor floor plan with a pedestrian tracking system using inertial sensors as a motion model along with Wi-Fi signals as an observation model. Yet another system called iFrame [20] uses multiple collaborating smartphones and incorporates three mobile sensing technologies—accelerometers to support dead reckoning, Bluetooth RSSI detection, and Wi-Fi RSSI detection—to improve the accuracies of indoor floor plan reconstruction. The iFrame models the unknown indoor map as a matrix and combines the three mobile sensing methods using curve fit fusion. By adopting shadow rates and anchor point analysis, iFrame extends the floor plan reconstruction from one room to an entire building.

Computer vision techniques have also been used for indoor map construction. For instance, JigSaw [21,22], extracts detailed geometry information about individual landmarks from images, and infers the structure and shapes of the hallway and rooms from inertial data to reconstruct the indoor floor plan using smartphones. Another system, Sextant [23], combines the photos obtained by smartphones with gyroscope information to draw an indoor map. These vision-based approaches reconstruct the indoor map with reasonable accuracy. Nevertheless, these computer vision techniques require considerable user intervention, with the procedure of image processing being complex and energy-intensive.

SLAM (Simultaneous Localization and Mapping) is a well-known technique used for learning and reconstructing maps in the mobile robotics domain [24,25]. SLAM estimates the 2D/3D locations and poses (i.e., orientations) of a robot; in addition, it estimates the location of landmarks near the robot from robot control and environment measurement parameters. SLAM employs various sensors including odometry, gyroscope, depth/stereo cameras, ultrasonic, and laser rangers. Luo et al. [26] designed an intelligent service robot that autonomously estimates the environmental structure and simultaneously detects some commonly recognized signs in the building. By employing multisensor data fusion techniques, i.e., covariance intersection and covariance union, developed to robustly associate pose and estimate signs, an information-enriched map is constructed. Furthermore, an improved alignment technique is applied to promote the mapping precision in a single simultaneous localization and mapping process using the posterior convenience. In general, SLAM requires specific hardware and incurs considerable computational costs to sense a sizable area and process complex collected data, which limits its widespread application.

Our proposed method uses the stable and unique sequence of magnetic anomalies as an index to select and merge all straight-line snippets partitioned from crowdsourced trajectories. As a result, the deviations and errors of single dead reckoning are reduced. Our system adopts an affinity propagation algorithm to cluster the crowdsourcing atomic magnetic trajectories into different groups using the DTW similarity criteria. Each group corresponds to one indoor path. All atomic trajectories inferred from odometry tracing belonging to the same cluster are fused to accurately estimate the paths. The scheme of multiple trajectory fusion effectively handles the inherent tracking errors from noisy sensors, which allows accurate indoor paths to be obtained.

## 3. Algorithm

The accuracy of floor plan construction in most current work is low because the indoor path is generally inferred by only one dead reckoning trajectory. The accuracy of the dead reckoning algorithm suffers from error accumulation due to sensor noise. To improve the accuracy of indoor floor plan construction, we propose a novel crowdsourcing indoor floor plan construction algorithm based on magnetic fingerprint. Our proposed algorithm accurately identifies multiple inferred trajectories walking along the same indoor path based on the steady and unique geomagnetic fingerprint associated with each individual path. By merging multiple trajectories associated with the same indoor path, the influence of occasional odometry tracing errors for indoor floor plan construction is greatly reduced.

### 3.1. System Architecture

**Figure 1:**
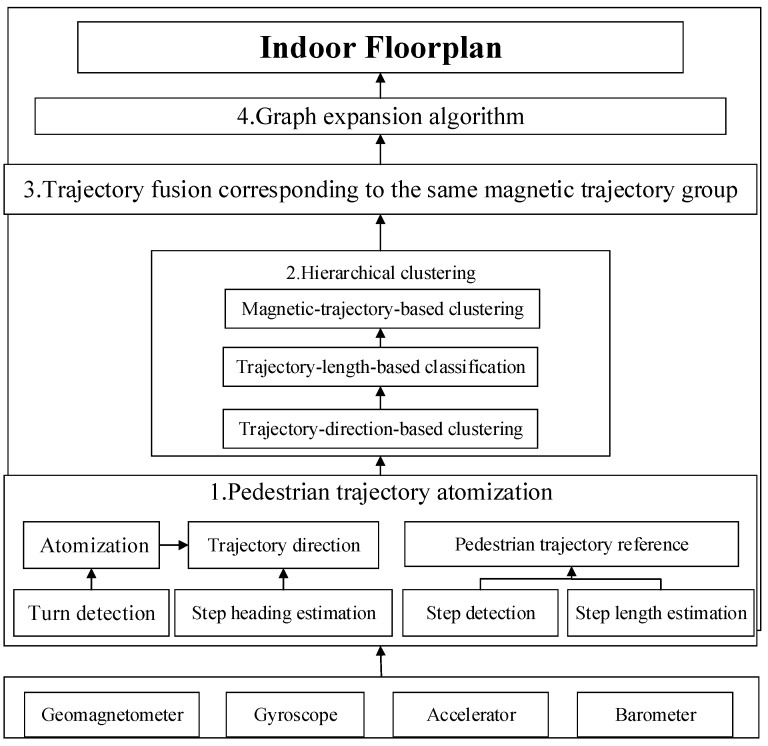
The architecture of the crowdsourcing indoor floor plan construction algorithm based on magnetic fingerprinting.

Figure 1 depicts the architecture of the crowdsourcing indoor floor plan construction algorithm based on magnetic fingerprinting. Our algorithm consists of four key steps:

(1) Pedestrian trajectory atomization, which is used to partition the long walking trajectories with multiple turns into short straight-line trajectories using a turn detection method.
(2) Grouping the atomized straight-line trajectories using a hierarchical clustering algorithm based on the constraints of trajectory direction, trajectory length, and DTW similarity criteria.
(3) Merging multiple trajectories belonging to the same group to obtain one main path.
(4) Reconstructing the floor plan using a graph extending algorithm.

In the following sections, more details of the proposed algorithm are given.

### 3.2. Pedestrian Trajectory Atomization

Complex indoor environments are characterized by large long trajectory combinations produced by various pedestrian walking paths. As these trajectories only partially overlap, it has proved difficult to take them as a whole to cluster and fuse into accurate indoor paths. One approach to solve this issue is to partition the long trajectory into short and straight-line snippets using a turn detection method, i.e., trajectory atomization.

**Figure 2:**
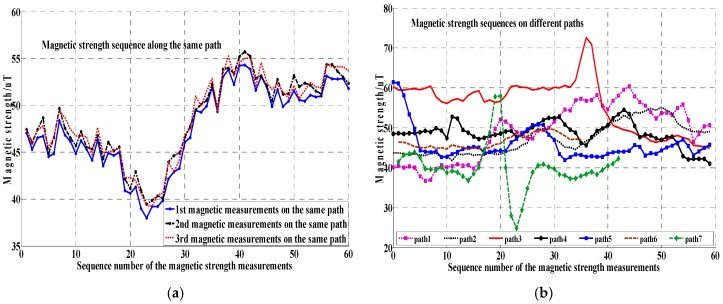
The magnetic trajectory comparison along the same and different indoor paths: (**a**) The steady and consistent magnetic sequence along the same path; (**b**) The different magnetic measurement sequences collected on different paths.

As described in [8], the geomagnetic anomalies caused by ferromagnetic construction materials (i.e., reinforcing steel bars) on the indoor path generally maintain steady and unique. The sequential geomagnetic data (called the geomagnetic trajectory) are collected when a user is walking along a specific indoor path and used as the representative feature of the corresponding path. As shown in Figure 2, the geomagnetic trajectories along the same indoor path are similar, while those along different indoor paths differ considerably.

To partition the long crowdsourcing indoor trajectories into regular and consistent short atomic trajectories, the indoor path turning point was used as the natural split location. All atomic trajectories are straight lines without any turning points in between. To accurately detect the turning point on a trajectory, we used a robust turn detection method that employs both an accelerator and a gyroscope, as Equation (1) shows.

(1) $$\left\{ \begin{array}{l} \theta =\sum_{i=1}^{m}\omega_{v}^{i}\Delta t\\ \omega_{v}^{i}=(\omega_{x}^{i},\omega_{y}^{i},\omega_{z}^{i})•({\bar{a}}_{x},{\bar{a}}_{y},{\bar{a}}_{z})/\sqrt{{\bar{a}}_{x}^{2}+{\bar{a}}_{y}^{2}+{\bar{a}}_{z}^{2}}\\ {\bar{a}}_{x}=\frac{1}{n}\sum_{j=1}^{n}a_{x}^{j},{\bar{a}}_{y}=\frac{1}{n}\sum_{j=1}^{n}a_{y}^{j},{\bar{a}}_{z}=\frac{1}{n}\sum_{j=1}^{n}a_{z}^{j} \end{array}\right.$$

where $\left(\omega_{x}^{i},\omega_{y}^{i},\omega_{z}^{i}\right)$ is the gyroscope observation of the smartphone in the carrier coordinate systems, and $\left(a_{x}^{i},a_{y}^{i},a_{z}^{i}\right)$ is the accelerator observation of the smartphone in the carrier coordinate systems. The vertical rotating speed $\omega_{v}^{i}$ is the dot product of the gyroscope observation vector $\left(\omega_{x}^{i},\omega_{y}^{i},\omega_{z}^{i}\right)$ and the unit vector of gravitational acceleration $({\bar{a}}_{x},{\bar{a}}_{y},{\bar{a}}_{z})/\sqrt{{\bar{a}}_{x}^{2}+{\bar{a}}_{y}^{2}+{\bar{a}}_{z}^{2}}$. To obtain the unit vector of gravitational acceleration, a sliding average method (n is the sliding window size corresponding to even steps, e.g., 4~6 steps) of the acceleration data is adopted to counteract the accelerator fluctuation caused by the pedestrian walking. The mean vector of the accelerator $\left({\bar{a}}_{x},{\bar{a}}_{y},{\bar{a}}_{z}\right)$ is approximate to gravitational acceleration, which points straight down to the gravitational center of the Earth. The rotation accumulation angle θ within a pre-defined sliding window (*m* is the corresponding window size of the time period) is used to determine whether a turn activity is happening when a pedestrian is walking on a certain path.

**Figure 3:**
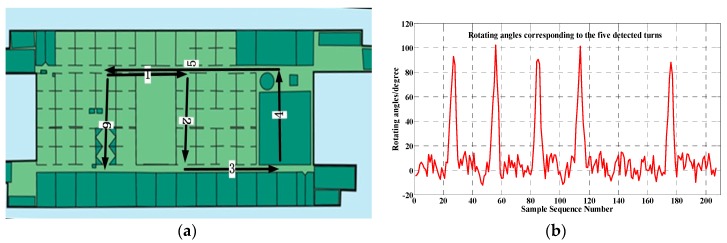
A typical experiment to evaluate the effectiveness of the turn detection method: (**a**) A long walking trajectory with five turns and six atomic trajectories; (**b**) The angle accumulation within the pre-defined sliding window corresponding to the trajectory.

The above-mentioned turn detection algorithm can accurately identify pedestrian turn activities. One typical evaluation experiment, shown in Figure 3, confirms the validity of the turn detection algorithm. All of the five turns along the long walking path (1->2->3->4->5->6) are correctly detected. The accumulation angles corresponding to the path turns are remarkably larger than other locations along the trajectory, as Figure 3b illustrates.

For the occasional turn activity of a pedestrian walking in the middle of indoor paths, the natural trajectory atomization will be further partitioned. However, the following clustering processing will detect these sparse atomic trajectories and remove them as invalid outliers.

In most cases, this turn-detection-based pedestrian trajectory atomization is accurate because the two neighboring corridors are approximately perpendicular to each other at the turn points. In rare cases, a turn with very small angle (i.e., less than 30 degrees) between two neighboring corridors is confused with user walking fluctuation, so the effectiveness of this pedestrian trajectory atomization is limited for turns with small angles. We leave it to our future studies to atomize a pedestrian trajectory with small-angle turns.

### 3.3. Hierarchical Classification

The indoor trajectory obtained from only one pedestrian walking suffers from the step length and heading error caused by the inertial and compass sensor noise. By identifying multiple crowdsourcing atomic trajectories corresponding to the same indoor path and merging them, the accuracies of the indoor trajectory and indoor floor plan are greatly improved. To reliably identify and correctly associate multiple inferred crowdsourcing trajectories walking on the same indoor path is the core point of our proposed indoor floor plan construction algorithm.

As mentioned in the above section, the magnetic measurement sequence along each indoor individual path is both steady and unique, and can therefore be used as the representative feature to cluster all the atomic trajectories.

#### 3.3.1. Hierarchical Clustering Architecture

**Figure 4:**
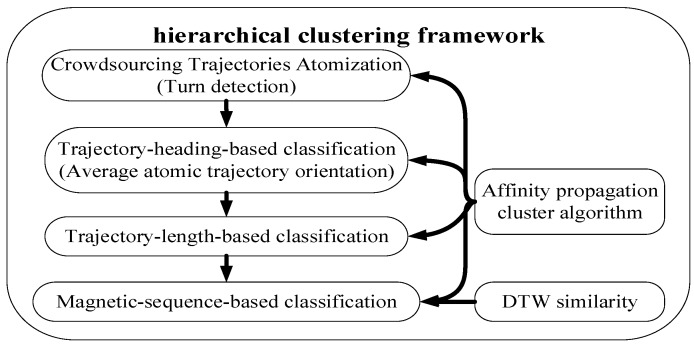
The hierarchical clustering framework used for the crowdsourcing atomic trajectories.

To improve the accuracy and speed of clustering in our proposed algorithm, we adopted a hierarchical clustering algorithm which comprises three classifiers: a trajectory-heading-based classifier, a trajectory-length-based classifier and magnetic-sequence-based classifier, as Figure 4 depicts.

#### 3.3.2. Trajectory-Heading-Based Classification

The atomic trajectory heading is defined in this paper as the mean compass heading of all detected steps along the atomic trajectory. Our previously proposed robust step detection algorithm was used [27].

Influenced by the geomagnetic anomalies caused by ferromagnetic construction materials (i.e., reinforcing steel bar) and the shaking activity during walking, an indoor compass heading fluctuates and deviates from the actual direction.

**Figure 5:**
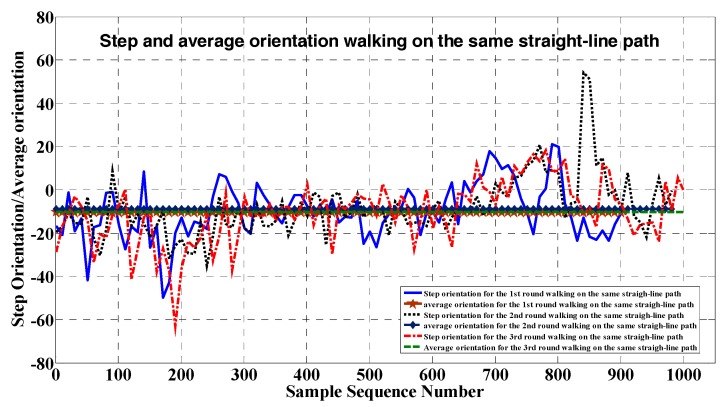
The comparison of the step and mean orientations walking along the same atomic trajectory.

To improve the atomic trajectory heading, we averaged the compass heading along this atomic path. As shown in Figure 5, the mean compass heading for repeatedly walking along the same indoor atomic trajectory remains stable, while the compass heading of each step fluctuates sharply because of non-uniform magnetic anomalies in the indoor environments.

The trajectory-heading-based classifier employs the mean heading of each individual atomic trajectory as a feature to group all the atomic trajectories into several clusters, which differs little from the actual orientations of the indoor paths.

**Figure 6:**
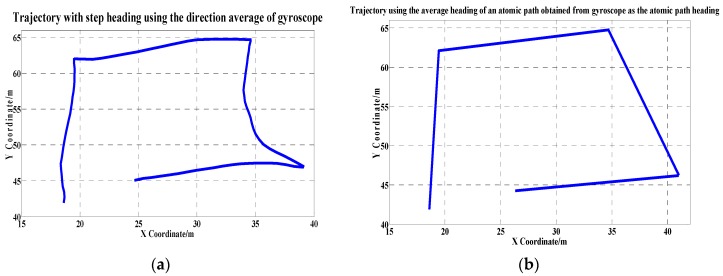

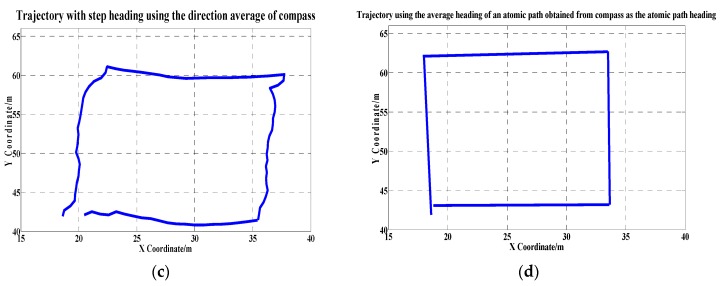
The trajectory inference using different heading estimation methods. (**a**) Trajectory using the heading of each step obtained from the direction average of gyroscope; (**b**) Trajectory using the average heading of an atomic path obtained from gyroscope as the atomic path heading; (**c**) Trajectory using the heading of each step obtained from the direction average of compass; (**d**) Trajectory using the average heading of an atomic path obtained from compass as the atomic path heading.

To further evaluate the effectiveness of our new method, we performed multiple walking experiments along a square path. The estimated trajectories with different heading estimation methods are shown in Figure 6.

First, we defined one step as a unit and calculated the average direction of a step obtained from the gyroscope as the step heading (Figure 6a). Different from taking a step as a unit, the trajectory heading in Figure 6b was calculated based on the unit of an atomic path, i.e., the average heading of an atomic path, obtained from the gyroscope, is regarded as the heading of the atomic path. The trajectory heading in Figure 6c was estimated based on the unit of a step using the compass. The trajectory heading indicated in Figure 6d was estimated on the unit of an atomic path using the compass. We obtained an accurate trajectory heading estimation by adopting our proposed orientation-averaging method along an atomic path, which combines the use of the compass, gyroscope, and accelerator.

In general, we did not know the prior number and headings of atomic trajectories for floor plan construction. We therefore employed an affinity propagation algorithm that allows for the clustering of all partitioned atomic trajectories. Using the affinity propagation algorithm, it is unnecessary to assign a specific value to control the group number for the affinity propagation algorithm. Nevertheless, if we can obtain the actual number of atomic trajectory headings, it is possible to obtain more accurate clustering results.

The trajectory-heading-based classifier effectively identifies the corridor orientations parallel or perpendicular to the building orientation.

#### 3.3.3. Trajectory-Length-Based Classification

After performing trajectory-heading-based clustering, we adopted the trajectory-length-based classifier to further group the atomic trajectories. The trajectory length was an estimation of the distance a user would walk on an atomic trajectory. The trajectory-length-based classifier aims to differentiate the indoor atomic paths with different length. It is based on the fact that many different indoor paths have the same orientation and different path lengths. We also used the affinity propagation algorithm for the trajectory-length-based classification.

#### 3.3.4. Magnetic-Sequence-Based Classification

After performing the trajectory-heading-based classification and trajectory-length-based classification, all the atomic trajectories were grouped into different clusters with different headings and lengths, which were further clustered using the magnetic-sequence-based classification to obtain the final classification corresponding to the same physical path. The magnetic-sequence-based classification was based on the observation that the magnetic measurement sequence along each individual indoor path is steady and unique. The atomic magnetic sequence was associated with the atomic odometry trajectory based on the same time base.

It is well-known that the DTW (Dynamic Time Wraping) [28] algorithm effectively measures the similarity between two temporal sequences which may vary in time or speed. To eliminate the influence of differing amounts of crowdsourcing magnetic data collected on the same path, caused by different walking speeds and different sampling frequencies, the magnetic-sequence-based classification algorithm leverages the DTW algorithm to calculate the similarity between two atomic magnetic sequences.

For two sequences of atomic magnetic trajectory data $M_{1}=\{m_{1}^{i}|i=1,2,\ldots ,n\}$ and $M_{2}=\{m_{2}^{j}|j=1,2,\ldots ,k\}$ (n and k are not necessarily equal), the similarity $d_{i,j}$ (i.e., the shortest accumulation distance) between the sequence $M_{1}$ and the sequence $M_{2}$ is calculated using the DTW algorithm, as shown in Equation (2).

(2) $$d_{i,j}=\min\;(d_{i-1},d_{i,j-1},d_{i-1,j-1})+dist_{i,j}$$

where $dist_{i,j}(i\in (1,n),j\in (1,k))$ represents the Euclidean distance between sample $m_{1}^{i}$ and sample $m_{2}^{j}$; $d_{n,k}$ represents the similarity of the two atomic magnetic data sequences.

After performing the trajectory-heading-based classification and the trajectory-length-based classification, we further adopted the magnetic-sequence-based classification to cluster the atomic magnetic sequences. The magnetic-sequence-based classification also employed the affinity propagation algorithm and the similarity criteria of DTW.

Considering the DTW time complexity $o(n^{2})$, introducing our proposed hierarchical clustering method enormously reduces the calculation cost. The similarity calculation is limited to only the much smaller clusters after the trajectory-heading-based classification and the trajectory-length-based classification. To further improve the calculation efficiency, the FastDTW algorithm [29] with o(n) complexity was used in this paper.

It should be pointed out that there are some overlapping atomic trajectories (Figure 4a). The atomic trajectories 1 and 5 overlap by around 50%. This situation usually occurs along a long corridor partitioned by several cross paths. These overlapping atomic trajectories were regarded as the same path and were merged.

### 3.4. Multiple Trajectory Fusion

After completing the hierarchical clustering operation, all the crowdsourcing trajectories were partitioned into different groups, which correspond to the indoor straight-line trajectory segments. Considering that the individual mobile trajectories are highly noisy due to sensor error accumulation, a multiple trajectory fusion scheme was introduced that merges all the atomic trajectories that belong to the same group to obtain the final accurate trajectory estimation.

**Figure 7:**
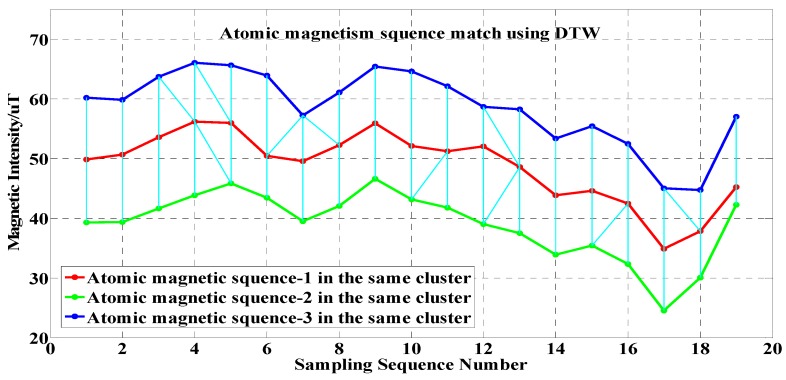
Multiple magnetic sequence match using Dynamic Time Warping (DTW) in the same cluster mapping geomagnetic data into physical location.

In the fusion process, each cluster center (the red curve indicated in Figure 7) acts as the index to map all the corresponding coordinate points associated with other atomic magnetic sequences (e.g., the blue and green curves in Figure 7).

After we performed the DTW-based magnetic sequence clustering, all points in the non-cluster-representative magnetic sequences in the same cluster were mapped to the points of the cluster-representative member.

**Figure 8:**
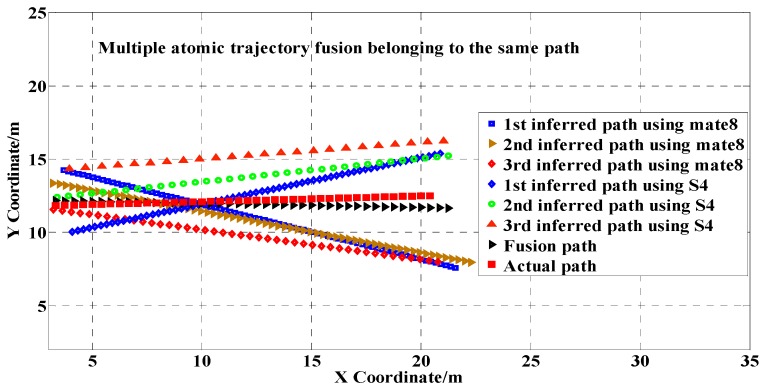
Multiple atomic trajectory fusion of trajectories belonging to a cluster.

All the mapped locations of the non-cluster-representative magnetic sequences were averaged with the corresponding location of the cluster center to obtain a location estimation of the path. Performing fusion of all locations of the cluster center produced an accurate indoor path estimation, as Figure 8 illustrates. Six atomic trajectories with different trajectory lengths and orientations were gathered by different users using two different types of smartphones (i.e., a Huawei Mate8 and a Samsung S4). All of the six raw atomic trajectories deviated from the actual path.

When performing the DTW-based multiple trajectory fusion, we found that the estimated path was similar to the actual path observed.

### 3.5. Floor Plan Construction Using Graph Extension Algorithm

In order to systematically generate a pathway map with path width information, we introduced the following expansion procedure.

First, we drew each fusion trajectory on a canvas, and then expanded it to a 0.5 m width (i.e., from line to shape) based on the distribution of the associated atomic trajectories. Occupied pixels are weighted proportionally according to their distances to the fusion trajectory, i.e., the closer the pixels, the higher the weight. Due to the multiplicity of atomic trajectories, the expanded atomic trajectories overlapped, and the weights of overlapping pixels were summed.

The expansion process continues until all atomic trajectories have been traversed and finally result in a fat pathway map. To delete the influence of some atomic trajectories with gross errors, a shrinking process is then applied to prune away those outer pixels whose weights are less than a certain threshold.

Considering that many atomic trajectories may be experienced more frequently than others, we adopted a dynamic weight threshold value. The dynamic weight threshold value of each pixel is set to a certain proportion of the maximum weight of the pixel’s neighborhood. The threshold proportion was set to 20% for the experiments in this paper. Finally, we removed isolated pixels and smoothed the edges of the resulting shrunk pathway map.

### 3.6. Complexity Analysis

The proposed indoor floor plan reconstruction system is based on the hierarchical clustering algorithm. We assume that the number of atomic trajectories is N, the average length of each magnetic sequence is M, the average length of each orientation sequence is L, the number of orientation clusters is K, the number of length clusters is R, the number of magnetic clusters is P, the average number of atomic trajectories in each cluster is N/K after orientation clustering, the average number of atomic trajectories in each cluster is N/R after length clustering, and the average number of atomic trajectories in each cluster is N/P after magnetic clustering.

The time complexity of the orientation clustering: The complexity of calculating the average orientation of all orientation sequences is Ο(N∗L)~Ο(N). The time complexity of AP (Affinity Propagation) clustering for orientation sequences is Ο(N∗N∗logN). As a result, the total complexity of the orientation clustering is Ο(N)+Ο(N∗N∗logN)~Ο(N∗N∗logN).

The time complexity of the length clustering: After orientation clustering, the time complexity of AP clustering for trajectory length is Ο(K∗(N/K)∗(N/K)∗log(N/K))~Ο(N∗N∗logN).

The time complexity of the magnetic clustering: After length clustering, the time complexity of calculating the similarity of all magnetic sequences in a cluster using FastDTW is Ο((N/R)^2∗M). The time complexity of AP clustering for magnetic sequences in a cluster is Ο((N/R)∗(N/R)∗log(N/R)). As a result, the total time complexity of AP clustering for magnetic sequences is R∗(Ο((N/R)^2∗M)+Ο((N/R)∗(N/R)∗log(N/R)))~Ο(N∗N∗logN).

The time complexity of fusing all trajectories in each cluster is O(P∗M).

The total time complexity of indoor floor plan reconstruction is Ο(N∗N∗logN)+Ο(K∗(N/K)∗(N/K)∗log(N/K))+Ο(N∗N∗logN)+O(P∗M)~Ο(N∗N∗logN).

Considering that there are limited atomic paths within a building and that the floor plan construction operation is run off-line, the calculation complexity of our proposed algorithm is acceptable.

## 4. Experiment and Analysis

### 4.1. Experimental Setup

**Figure 9:**
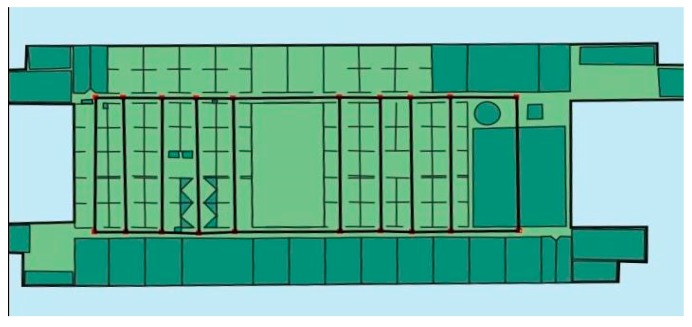
The layout of the experimental area on the 7th floor of the Institute of Computing Technology, Chinese Academy of Sciences.

To fully evaluate the performance of our proposed floor plan building algorithm, we conducted practical experiments on the 7th floor of the Institute of Computing Technology, Chinese Academy of Sciences. The experimental area is approximately 70 × 40 m^2^, with the indoor layout shown in Figure 9.

During the experiment, we held a smartphone in hand, in front of our chests, and walked along the corridors. We collected data using Android smartphones (a Huawei mate 8 with 8 core 2.3 GHz processor and a Samsung S4 with 4 Core Processor). The smartphones are equipped with a 3-axis accelerometer, a gyroscope sensor, and a 3-axis Magnetic field sensor. The smartphone periodically collected the data generated from the magnetometer, gyroscope, and accelerometer with 2 Hz sampling frequency. We walked at a constant speed and collected data on twelve different paths. We uniformly selected ten test points on each path and recorded their actual locations. When the testers passed these test points, they pushed the smartphone screen to trigger our developed APP to obtain the actual location of the corresponding test point. All these selected test points were used to evaluate the map construction accuracy based on the cumulative distribution function.

To quantify the performance of the proposed algorithm, we performed simulations with various parameters. The simulation was conducted on a Windows 7 desktop computer with 4 × 3.2 GHz CPU and 16 GB RAM.

### 4.2. Performance Evaluation

#### 4.2.1. Floor Plan Construction

**Figure 10:**
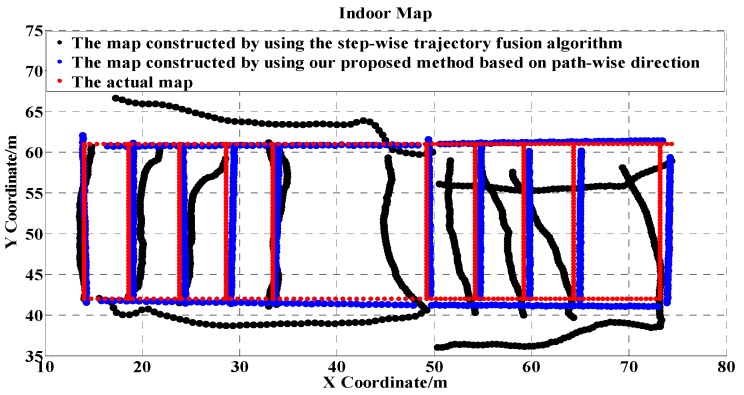
The constructed floor plan comparison by using our proposed algorithm and by adopting the step-wise trajectory fusion algorithm.

Figure 10 shows the constructed floor plan by adopting our proposed algorithm. As a comparison, the figure also shows the constructed trajectories by adopting a step-wise trajectory fusion algorithm which fuses the individual paths based on step-wise direction, i.e., taking the raw heading of each individual step as the path direction. Due to the fluctuation of heading caused by environmental factors, the trajectories inferred by the step-wise trajectory fusion algorithm greatly deviated from the actual path.

**Figure 11:**
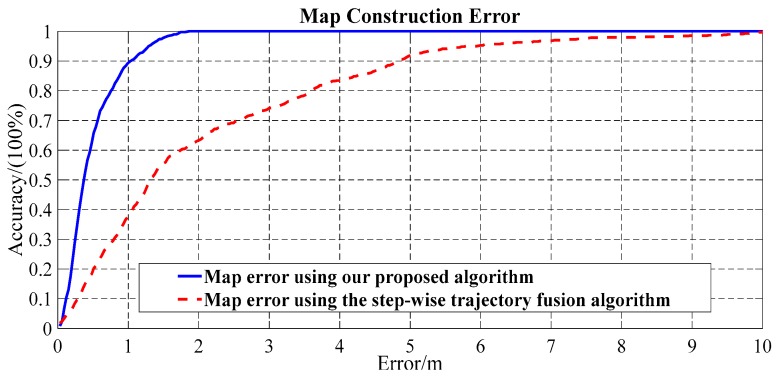
Floor plan construction error comparison using our proposed algorithm and the step-wise trajectory fusion algorithm.

Figure 11 compares the map construction error in the cumulative error distribution (CDF) form from using the proposed algorithm and from adopting the step-wise trajectory fusion algorithm. Our proposed algorithm achieved 1 m map construction error with about 90% confidence, while the step-wise trajectory fusion algorithm achieved 1 m map construction error only with 40% confidence. Table 1 shows that the average map construction error using our proposed algorithm is 0.48 m, which is 1.59 m less than the step-wise trajectory fusion algorithm. Compared with the step-wise trajectory fusion algorithm, the standard deviation of the construction error using our proposed algorithm is also substantially smaller.

#### 4.2.2. Calculation Costs

Table 2 lists the calculation costs resulting from running our proposed algorithm on a PC with 4 × 3.2 GHz CPU and 16 GB RAM. It takes approximately 104 s for our proposed algorithm to complete the floor plan construction, with most of the time used incurred by the clustering operation. However, considering the algorithm is used for off-line map construction, the requirement of calculation cost is not critical.

## 5. Conclusions

This study has revealed some useful insight on low-cost indoor map construction using crowdsourcing data generated by the compass, gyroscope, and accelerator embedded in smartphones. Compared with the existing floor plan construction methods, in this work, we take advantage of the stable and unique feature of magnetic anomalies caused by modern building ferromagnetic construction materials, and take the magnetic anomaly sequence as a unique index to group and merge all crowdsourced trajectories. Because the geomagnetic field is globally distributed, our proposed floor plan construction algorithm is pervasive. Another advantage of our system is that no additional hardware such as a laser rangefinder or wheel encoder is required.

To obtain accurate floor plan construction results, our system first adopts an affinity propagation algorithm to cluster the crowdsourcing trajectories, and then merges the atomic trajectories (inferred from odometry tracing) belonging to the same cluster to accurately estimate the paths. The fusion scheme of multiple trajectories effectively handles the inherent tracking errors from noisy sensors, which allows accurate indoor paths to be obtained. Furthermore, we proposed a novel hierarchical clustering algorithm and adopted it to cluster the crowdsourcing data into multiple groups with high calculation efficiency.

The evaluation results demonstrate that our proposed algorithm successfully constructs indoor floor plans with 0.48 m accuracy, which outperforms other existing floor plan methods and could benefit the location-based services which lack indoor maps.

Future studies testing large-scale applications should greatly benefit from our proof-of-principle study.

## Figures and Tables

**Table 1:** The map construction error by using the proposed algorithm and the step-wise trajectory fusion algorithm.

Algorithm	Mean Construction Error	Standard Deviation of Construction Error
Our proposed method	0.48 m	0.37 m
The step-wise trajectory fusion algorithm	2.07 m	1.99 m

**Table 2:** The calculation time cost by our proposed algorithm.

Algorithm	Clustering Operation	Merging Operation	Total Calculation Time
Our proposed method	101.21 s	2.12 s	104.37 s
